# Revisiting characteristics of oncogenic extrachromosomal DNA as mobile enhancers on neuroblastoma and glioma cancers

**DOI:** 10.1186/s12935-022-02617-8

**Published:** 2022-05-25

**Authors:** Mohsen Karami Fath, Nastaran Karimfar, Andarz Fazlollahpour Naghibi, Shahriyar Shafa, Melika Ghasemi Shiran, Mehran Ataei, Hossein Dehghanzadeh, Mohsen Nabi Afjadi, Tahereh Ghadiri, Zahra Payandeh, Vahideh Tarhriz

**Affiliations:** 1 Department of Cellular and Molecular Biology, Faculty of Biological Sciences, Kharazmi University, Tehran, Iran; 2 Faculty of Veterinary Medicine, Islamic Azad University, Tabriz Branch, Tabriz, Iran; 3 Faculty of Medicine, Islamic Azad University, Sari Branch, Sari, Iran; 4 School of Medicine, Kerman University of Medical Sciences, Kerman, Iran; 5 Department of Biology, Faculty of Sciences, Central Tehran Branch, Islamic Azad University, Tehran, Iran; 6 Department of Biology, Faculty of Sciences, Shahid Chamran University, Ahvaz, Iran; 7 Khoy University of Medical Sciences, Khoy, Iran; 8 Department of Biochemistry, Faculty of Biological Science, Tarbiat Modares University, Tehran, Iran; 9 Department of Neuroscience and Cognition, Faculty of Advanced Medical Sciences, Tabriz University of Medical Sciences, Tabriz, Iran; 10 Shefa Neuroscience Research Center, Khatam Alanbia Hospital, Tehran, Iran; 11 Neurosiences Research Center, Tabriz University of Medical Sciences, Tabriz, Iran; 12 Department Medical Biochemistry and Biophysics, Division Medical Inflammation Research, Karolinska Institute, Stockholm, Sweden; 13 Molecular Medicine Research Center, Biomedicine Institute, Tabriz University of Medical Sciences, Tabriz, Iran

**Keywords:** Cancer, Extrachromosomal circular DNA, Drug resistance, Oncogene, Amplification

## Abstract

Cancer can be induced by a variety of possible causes, including tumor suppressor gene failure and proto-oncogene hyperactivation. Tumor-associated extrachromosomal circular DNA has been proposed to endanger human health and speed up the progression of cancer. The amplification of ecDNA has raised the oncogene copy number in numerous malignancies according to whole-genome sequencing on distinct cancer types. The unusual structure and function of ecDNA, and its potential role in understanding current cancer genome maps, make it a hotspot to study tumor pathogenesis and evolution. The discovery of the basic mechanisms of ecDNA in the emergence and growth of malignancies could lead researchers to develop new cancer therapies. Despite recent progress, different aspects of ecDNA require more investigation. We focused on the features, and analyzed the bio-genesis, and origin of ecDNA in this review, as well as its functions in neuroblastoma and glioma cancers.

## Introduction

Extrachromosomal DNA (ecDNA), owning a size from 1 to 3 megabytes and a median of 1.26 megabytes, is visible under light microscopy. It carries complete genes. EcDNA has first identified in neuroblastoma (NB) cells as a pair of tiny chromatin structures known as double minutes (DMs) [1, 2]. Jack et al. found that identical sister minutes appear as two spherical chromatin masses joined by chromatin fibers in scanning electron microscopy (SEM) analysis of DMs [3]. In a study by Turner et al. the structural modeling and cytogenetic investigation of the whole-genome sequencing (WGS), the existence of ecDNA in diverse malignant samples and cancer cell lines is confirmed [4]. Considering its size, one or more complete genes, such as oncogenes, can be found in ecDNA. For instance, N-MYC proto-oncogene (MYCN), a particular deregulated oncogene of poor prognosis cancers, was primarily found in neuroblastoma samples ecDNAs [5].

The amplification of oncogenes as a common feature of cancer genomes [6] causes proto-oncogene overexpression. It has been discovered that removing ecDNA can reduce oncogene amplification on ecDNA, and consequently restore the tumor phenotype [7]. Self-repeating arrays on a chromosome homogeneously staining regions (HSR) and multiple individual ecDNA copies are two types of supernumerary gene copies. EcDNA not only can be formed during chromothripsis or other genome reshuffling events but also it can make up many coding and non-coding chromosomal distal regions. Amplified DNA accumulates more internal rearrangements and mutations, causing adaptive changes like resistance against targeted therapy. As HSRs, ecDNA reintegration into chromosomes can also operate as a general driver of genome remodeling and a key genomic characteristic in a variety of cancers via probable induction of intrachromosomal amplification [1, 7, 8]. Fortunately, advances in biotechnology have made it possible to recognize and produce ecDNA using different approaches. These technologies allow researchers to highlight the structure of ecDNA and speculate its part in cancer [2]. Since, several studies have revealed a remarkable role of ecDNA in the carcinogenesis of neuroblastoma, glioblastoma (GBM), ovarian cancer (OC), colon cancer, and breast cancer [9], herein, after the structural and functional introduction of ecDNA, we will focus on its contribution in the pathobiology of cancers such as neuroblastoma, and glioma cancers.

## Biogenesis of ecDNA

**Fig. 1:**
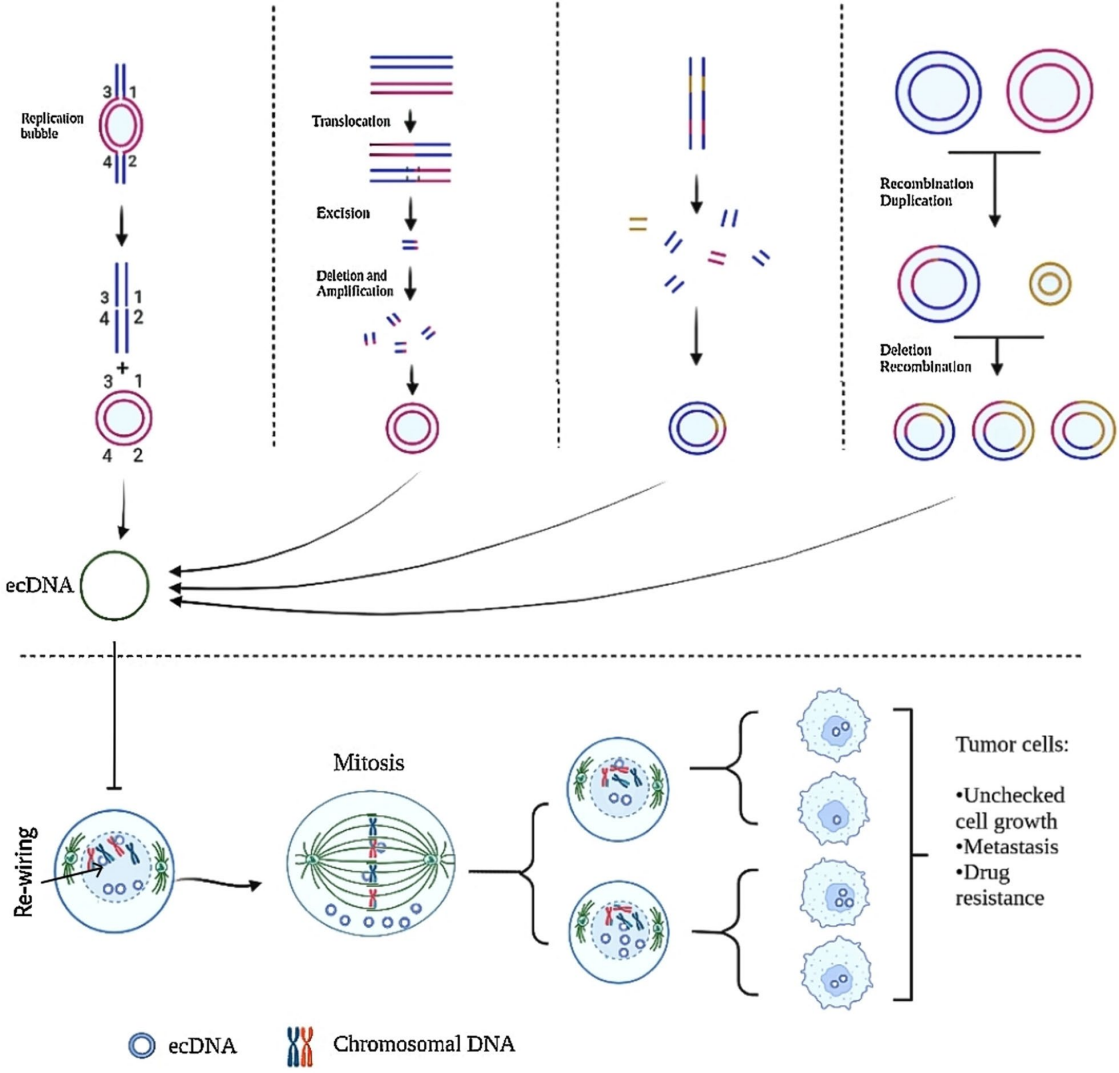
Biogenesis and genesis of ecDNA (up) role of ecDNA in cancer

Endogenous DNA damage (e.g., DNA replication stress), external stress (e.g., carcinogens and infections), and abnormalities in the DNA damage repair machinery are all biological sources of ecDNA generation [10]. EcDNA and HSR of chromosomes could be produced during gene transcription, in favor of the genome’s complexity and plasticity [5]; however, the exact mechanisms of ecDNA biogenesis remain unknown. EcDNA can be created through various cell cycle events. One of the most widely used models of ecDNA construction in which replication fork stalling results in replication fork collapse, and then causes the replication bubble to fall off the chromosome, interconnect, and form the episome is one that recommends their autocatalytic recombination which leads to DM formation (Fig. 1) [7, 11].

The formation of Myelocytomatosis (MYC)-containing DMs in acute myeloid leukemia (AML) is recent proof of the episome concept. Based on the results of fluorescence in situ hybridization (FISH) analyses on metaphase chromosomes of bone marrow samples by Storlazzi et al. 23 of the 30 AML cases with MYC-containing DMs had a deletion of the MYC gene area on chromosome 8. DM linker sequences match those of chromosome 8, in which the MYC gene is deleted, which indicates that the MYC gene resides on chromosome 8 and comes from DM [12]. Later, it was hypothesized that the episome model could be used to evaluate solid tumors, for instance, NB and small cell lung cancer (SCLC) [13]. Sequencing of cancer genome resulted in introducing a novel model of ecDNA construction called chromothripsis in which abnormal isolation of chromosome and then its termination to a micronucleus causes more replication stress, DNA breakage, and poor repair because of limited access to replication proteins [14]. Therefore, by considering the association of small segments of the chromosome at hundreds of kilobases distant from contigs in the DMs, and the association of different junctions with fusions among non-contiguous sequences in the normal reference genome [15], the chromothripsis concept could be linked to the existence of rearrangements in the course of the development of DMs. Given that miscellaneous genetic and molecular analysis on SCLC cell line (GLC1), it has been shown that ecDNA endures multiple evolutionary steps. Indeed, the presence of multiple DM subpopulations with a variety of shared structural variations (SVs), including single chromosome and/or combinations of various chromosomes produced through undergoing a sequence of recombination, deletions, and duplications, has been proposed. This processing manner caused several chromosomal origins such as chromosome 1, 8, or 21 for single-chromosome ancestral DMs in the GLC1 cell line [16]. In theory, chromosomes 1 and 8 were recombined to form a new DM, but they underwent various deletions and recombinations with that new DM, resulting in several subpopulations of DM [16]. Given the “episome paradigm” of gene amplification [17], the chromosomal arm's submicroscopic circular episome was retained and multimerized to form larger DMs. Once a circle is incorporated into a chromosome arm, the breakage-fusion-bridge cycle (BFB) is induced, ensuing in chromosomal HSR. This theory has been examined by employing a plasmid with a replication initiation region (IR) and a nuclear matrix (scaffold) attachment region (MAR/SAR), both of which were essential for replication initiation. When such plasmids were transfected into COLO 320DM cells from human colorectal cancer, DMs and/or HSRs were produced in stable transformants that were morphologically indistinguishable from those found in malignant cells [18]. Dihydrofolate reductase (DHFR), c-MYC [19], and -globin IR [20] were used to identify the minimum sequence required for effective amplification, and such core IR contains a variety of sequence components required for replication starts. This system was used to investigate the mechanism of gene amplification. The IR/MAR-containing circular plasmid DNA was multimerized into enormous circles, with the sequences organized in tandem repeats [18]. on the condition that the largest circle grows enough, it can be visible as DMs under light microscopy [21]. The IR/MAR plasmid’s tandem repeat was subsequently incorporated into the chromosomal arm, where it effectively kicked off the HSR-producing BFB cycle [22]. As replication begins at around 100 kbp intervals, the IR/MAR regions that allow gene amplification are dispersed across the human genome [23]. As a result, at least a fraction of the multiple tiny extrachromosomal circular DNA elements (eccDNAs) created from the chromosomal arm should be amplified in the same way as the IR/MAR plasmid. Thus, along with plasmid, IR/MAR amplification, DNA of transfected cells was also amplified [18], implying that extrachromosomal DNA recombination is common. Innate DMs/ecDNA are an assortment of sequences that originate from diverse chromosomal regions [24]. The co-amplification of extrachromosomal circles and distant enhancer sequences of the oncogene could be expected to intensify theoncogene expression, too [25]. Likewise, the amount of IR/MAR gene amplification in normal cells is different in malignant cells, as well as tumor cell lines. This could be due to the fact that DM/ecDNA and/or gene amplification are merely found in a few types of cancer cells [4]. Moreover, specific kinds of cells tend to produce IR/MAR sequences. [26]. It should be noticed that multimerization of the episome/eccDNA containing the IR/MAR sequence creates larger and more complex DMs/ecDNAs [27]. The process that causes the chromosomal arm to produce an initial tiny circle was addressed and replicated in culture by a model system [26]. In this system, chromothripsis occurs when a specific chromosome is abruptly fragmented, re-ligated, and then distributed by the rearrangement of numerous fragments [27]. The fragmentation of a specific chromosome may occur as a consequence of a rupture in the nuclear membrane of the micronuclei [28]. In these micronuclei, replication [29] and transcription [30] are faulty. The fragment is re-ligated, resulting in a significant number of circular molecules [14]. The circles having IR/MAR would be enhanced among these circles, as described above. Aiming to replicate the procedure of DMs/ecDNAs synthesis, hybrid human-rodent cells were cultivated. Because human centromere in such hybrids was failing, human chromosome was selectively integrated into micronuclei. The human chromosome was then removed once the micronuclear material was broken. Afterward, stable rat chromosomes possessed multiple acentric stable DMs/ecDNA with a mark of human genome, namely Alu [31]. The gene amplified property of circular plasmid DNA containing the IR/MAR makes it an ideal tool for human cancer genetic studies and recombinant protein manufacturing [32, 33].

## Characteristics and intra-cellular behavior of ecDNA

Nowadays, using novel sequencing tools, the circular form of ecDNA plus its formation in the extrachromosomal sites are widespread [2, 34, 35]. Besides full genes, ecDNA also contains non-coding sequences such as promoters and enhancers [25]. Being acentric, ecDNA segregates unequally during cell division so that daughter cells can receive two-folded ecDNA particles compared to the original cell [36]. This unequal ecDNA division causes the disparate distribution of genetic material and consequently can increase oncogene copy number along with intratumoral heterogeneity in favor of tumor formation and tumor adaptation, respectively [37]. Due to its less nucleosomal compactness than chromosomal DNA (chrDNA), ecDNA packs carrying acetylation of histone H3 at lysine 27 (H3K27ac), a powerful enhancer, are more prone to transcription. Thus, in the presence of circular ecDNAoncogene, expression significantly is elevated and promoted intra-tumoral heterogeneity by inducing chromatin rearrangement [35] (Table 1). In a study on three thousand malignancy cases, a link between oncogene amplification and ecDNA structure was also discovered. They have claimed that the individual genetic background together with the characteristic of the surrounding environment determines the destiny of cancer genomes [4, 38]. By interacting with remote areas of chromatin, ecDNA can regulate the expression of the distant genes [2]. It seems that during ecDNA formation enhancers are co-selected with oncogene coding regions [25]. Sometimes, ecDNA produces a chimeric sequence via the combination of DNA from several discrete chromosomes. Tumor suppressors incorporate DMs into the cytoplasmic micronucleus and then the aggregated DMs form micronuclei, which could be removed, re-corporated into the genome, or converted to HSRs [39]. Occasionally, structural flexibility of DMs, for example; reduction in the oncogene copies fade or abolish its carcinogenic effects [40]. Despite its effect on the ecDNA, hydroxyurea therapy did not decrease the number of oncogene copies on HSRs, demonstrating that oncogene expression is not unsteady on HSRs [41]. Glioblastoma cells, for example, exhibit significant quantities of oncogenic EGFRvIII in their ecDNA [42]. Under erlotinib therapy on glioblastoma cells (GBM39) doing structural studies of EGFRvIII amplification, ecDNA reintegrated into HSR. Interestingly, when erlotinib was stopped, the ecDNA amplicon appeared again [4]. Furthermore, adjusting EGFRvIII levels in ecDNA can improve glioblastoma cell resistance to tyrosine kinase inhibitors [43]. Whilst chromosomes are separating, the DM aggregates can not corporate into the chromosome creating micronuclei [44].

**Table 1:** Characteristics of ecDNA

	Abbreviation name	Number of strand(s)	Size	Origination	Sequence feature	Cancer association machanism	Refs.
Extrachromosomal small circular DNA	eccDNA	Single or double	< 1 Mb. invisible under microscope	Telomere circle, spcDNA, miDNA, episome	Small regulatory RNA	tumurgenic through selective teleomeric extension, modifying geneome stability	[9, 66, 71, 107]
Extrachromosomal DNA (double minutes)	ecDNA	Double	1–3 Mb, visible under microscope	BFB cycle, translocation/deletion amplification, episome and chromothripsis	Oncogene amplification, regulatory regions, no centromeres or telomeres	Oncogene amplification, chromosome, rearrangement Gene fusions, epigenetic/Histone, modification, nucleosome accessibility, signaling pathways regulation intra-tumoral heterogeneity autophagy, metastasis and invasiveness, senescence antitumor immunity	[1, 9, 25, 108]

In general, a large amount of damaged DNA was aggregated. There is a DNA transfer from the nucleus into the cytoplasm through the interphase nuclear barrier [45] or nuclear budding or nuclear membrane breakage [46]. This is significant because cytoplasmic chromatin activates cGAS-STING-related inflammatory response [47].

## Main methods in ecDNA discovery research

EccDNA imaging can be done with specific three-dimensional high-resolution microscopic equipment named 3D-SIM that has been developed in recent years [2]. Because of the limited resolution of light microscopy, electron microscopy is not sufficient to visualize the tiny structure of eccDNA. To tackle this difficulty, light microscopy has been modified using scanning and transmission electron microscopy dyes to detect ecDNA signals in cells. To detect eccDNA, some researchers employed a photoelectric approach overlaying confocal light microscopy and SEM signals [2]. As a preliminary technique, density based centrifugation using cesium chloride was first employed in 1958 to separate 14 N and 15 N DNA [48] and now because of large testers’ prerequisites and low accuracy in the detection of eccDNA, this technology is underutilized. Another detection way, an assay of transposase accessible chromatin (ATAC), is a transposase-mediated imaging method that works by in situ imaging, cell sorting, and deep sequencing of the genome. The combined ATAC with flow cytometry provided the possibility of quantitative analysis [49]. This approach is currently being utilized to visualize the accessibility of ecDNA in metaphase chromatin [2]. FISH as a cellular genetic study tool targets nucleic acid in fixed cells. It finds chromosomal location of individual genes, along with mutations, and analyzes the chronological and local expression of genes using fluorescently labeled DNA or RNA probes. Additionally, FISH can identify eccDNA in assigned samples through fluorescent probes. In contrast to the low read length of next generation sequencing (NGS) technique that produces read mapping mistakes and ambiguities [50], third-generation sequencing, (TGS) a new DNA sequencing technique is really promising. Considering the normal large length and multichromosomal composition of ecDNA constructing a full-length ecDNA sequence is challenging. TGS technology has made a considerable improvement in the read length issue, so that it can successfully detect full-length ecDNA sequences [51]. However, direct observation of eccDNA under a microscope is sufficient to prove its circular or non-circular shape, investigators also use two-dimensional electrophoresis to indirectly confirm the round form of eccDNA. In 1970, 2-DE was proposed for the first time [52]. Electrophoresis as a common molecular biology research technique differentiates elements based on the molecular weight and electrical charges. It is also capable to distinguish circular and linear DNA since their structures are fundamentally dissimilar. Scientists have recently developed Circle Sequencing, a sensitive, large-scale circular DNA detection approach based on plasmid manufacturing knowledge [53, 54].

Chromatin immunoprecipitation (ChIP) seq technique can track coding DNA particles in the complete genome using two ChIP and NGS technologies. The ChIP-Seq works based on the following principle: The DNA fragments are first enriched and purified using ChIP, following by library building and NGS analysis of the enriched DNA fragments. Researchers have pinpointed millions of sequence tags on the genome to characterize the DNA segments interacting with histones and transcription factors throughout the genome [55]. Given the significant monomethylation of histone H3 at lysine 4 (H3K4me1) and H3K27ac modifications in plasma eccDNA, the application of ChIP-seq could be advantageous for eccDNA investigations [56]. The next method is 4C-seq, which is a distinguished method for sophisticated investigation of the human chromatin as well as ecDNA [2, 57]. The method is based on the DNA and DNA-bound proteins cross-linkage, then digestion and ligation. After crosslinking, the DNA is purified. To generate a 4C library, the second round of digestion and ligation is done. The circularized and amplified content of 4C-seq PCR is detectable using NGS 4C-seq that can examine the interaction of a single chromatin segment with multiple chromatins in greater detail [58]. In mammalian cells, proximity ligation assisted ChIP seq (PLAC seq) is known as a quick, sensitive, and cost-effective approach for mapping long-distance chromatin connections. It is mostly utilized to figure out how different chromatin regions interact together. The procedure works by gluing cells together with formaldehyde. In situ, the biotin-labeled nucleotides are filled in and joined. The chromatin is trimmed after the nucleus is lysed. Transcription factors or histones oriented- antibodies are used to immunoprecipitate the soluble chromatin fraction. Finally, the biotin-labeled DNA that corresponds to the linker is enriched, followed by library preparation and paired-end DNA sequencing [59, 60]. The PLAC-seq was utilized to map genome-wide 3D chromatin connections anchored at DNA bound by specific histone modification (H3K27) [2].

Amplicon Architect's eccDNA analysis employs WGS to rebuild the accurate construction of focal genome modifications, and it has been meticulously endorsed under virtual versus real conditions [61]. Therefore, it can use short-read data to reconstruct probable ecDNA and other focused amplicon structures and offers interactive exploration of different configurations. Because of the size of ecDNA, single-molecule assembly is insufficient for ecDNA refurbishment. As a result, amplicon architect reconstruction on the low-cost short-read data can be utilized as a frame for the assembling of larger reading frames. Analyses of more than sixty malignant specimens with viral contamination using amplicon architect revealed a remarkable quantity of amplified DNA with exclusive physical properties indicating both human-virus extrachromosomal DNA [61]. The assessments of reassembled amplicons in multiple pan-cancer datasets emphasize the crucial role of ecDNA in the emergence of multifarious readjustments and localized exaggeration is seen through the cancer subtype spectrum. The fundamental trigger of focal copy number amplification (FCNA) is an amplicon reconstructor of ecDNA, which facilitates gene amplification, fast tumor progression, and rewires regulatory circuits [62]. Understanding FCNA's structure is the first step in the discovery of its biological outcomes and the underlying mechanism. The amplicon reconstructor approach can resolve FCNA with single-nucleotide resolution by combining optical mapping with NGS. Researchers have discovered the complicated ecDNA structure, break-fusion bridges, and other complex rearrangements in some cancer cell lines using the amplicon reconstructing of CNAs [62]. EcSeg is a U-Net-based platform for classifying 4′,6-diamidino-2-phenylindole (DAPI) signals, identifying and quantifying ecDNA, and combining FISH data to elucidate oncogene amplification on ecDNA and chromosomes. To calibrate and count ecDNA, ecSeg classifies every graphical signal in DAPI- and FISH-stained pictures into one of four classes: cytoplasm, nucleus, chromosome, and ecDNA, and computes the coupled signal of ecDNA. ViFi is a technique for the detection of viral genomes among mRNA sequences [63]. ViFi employs a reference and a phylogeny-based method to identify virus reads. The mappability score of the measurements is also used by ViFi to exclude false positives and mixed detection. The high specificity of this technique leads to detect integrated viruses despite their great modifications. ECdetect is an automated image analysis software detecting DAPI dye radiation from ecDNA in metaphase and is used in conjunction with whole-genome sequencing for cytogenetic analysis [64]. The software was applied to measure ecDNA in numerous cells from different cancer types, tumor cell lines, and noncancer control cell lines [4]. ChIA-PET (chromatin interaction analysis by paired-end tag sequencing) and chromatin interaction analysis with droplet sequencing are other tools for the research on oncogenic sequences. In 2021, Zhu et al. showed that known ecDNAs can be identified by an outline of solid intramolecular and intermolecular interactions along genome-wide chromatin. According to their findings interaction areas have many of the same properties as super-enhancers, which are known to trigger high-level oncogene transcription in many tumor types. Moreover, these results suggest that ecDNAs operate as mobile transcription-amplifying elements in human malignancies, in addition to oncogene amplification [65].

## The mechanistic actions of ecDNA in cancer

ecDNAs have the main role in various cellular pathways such as oncogene amplification, chromosome rearrangements and cell processes, which are related to cancer development including metastasis/invasion, autophagy, drug resistance, medication response, and clinical outcome [66]. Oncogene amplification is a key factor in carcinogenesis. This amplification occurs at ecDNA or chromosome HSR structures. Oncogene amplification in ecDNA significantly enhances overall oncogene expression, which is common in primary and metastatic cancers regardless of treatment method. EcDNA has the potential to reintegrate into chromosome HSRs and/or impact the accessibility of DNA for oncogene expression stabilization [43]. Oncogene amplification is the main source of ecDNAs as long as the breaking sites existed between the telomere and the hairpin break. It’s worth noting that the enhanced expression of ecDNA-encoded genes and their accumulation form a positive feedback regulatory loop [67]. Downregulation of ecDNA genes has also been linked to a reduction in the incorporation of ecDNA into cytoplasmic micronuclei. In light of these findings, a molecular link between ecDNA accumulation, oncogene hyperactivity, drug resistance of the cancerous cells, and cancer therapy resistance could be in play [4, 67, 68]. The common mechanism of medication resistance such as methotrexate (MTX) resistance can be caused via DHFR gene amplification [69]. According to Morales et al., MTX increased the copy number of the DHFR gene dramatically in colon cancer HT29 cells, whereas extrachromosomal DMs caused dramatic increases in the number of copies of the DHFR gene. A decrease in drug resistance capacity is also associated with the loss of the DHFR amplicon in MTX-sensitive cells when MTX is withdrawn. Those with drug resistance caused by gene amplification could potentially benefit from a second treatment round [70]. These results also suggested that by reducing extrachromosomal oncogene amplification, the homologous recombination mechanism could be used to increase chemotherapy effects [71]. Amplification of the proto-oncogene HER2, a receptor tyrosine kinase (RTK) from the epidermal EGFR family, was found in roughly 20% of breast cancers, while DM amplification was seen in nearly 30% of HER2-positive tumors. Although these cancers can react to direct HER2 treatments, they frequently develop resistance and regress [72]. Even when resistance to anti-HER2 treatment is acquired by lack of HER2 protein expression, the number of DMs containing HER2 is preserved in various models, implying that un-expression of HER2 protein due to the loss of DMs containing HER2 cannot be considered as the main mechanism of anti-HER2 therapy resistance [73]. EcDNA from solid tumors like medulloblastoma, leukemia, myeloma, and gastric cancer has been found to include gene fusions. Graux et al. have proposed a novel mechanism for tyrosine kinase activation. In this scenario, it has been proposed that a gene fusion between NUP214 and ABL1 genes produced ecDNA in acute lymphoblastic leukemia T-cell. It has also been demonstrated that the BCR-Abl1 fusion gene and the (9; 22) (q34; q11) translocation can be amplified on ecDNA, particularly in chronic myelogenous leukemia after imatinib treatment [74–76]. Epigenetic changes control the accessibility of ecDNA and chromatin and are responsible for a variety of biological activities. Chemical modification of chromatin, gene restoration, chromatin interaction, and topological re-instauration are examples of these alterations [77, 78]. For example, activating histone markers (H3K4me1 and H3K27ac) are more abundant in ecDNA than repressive histone markers (H3K4me1 and H3K27ac) (H3K9me3 and H3K27me3). On the ecDNA of glioblastoma cells, metaphase analysis also indicated increased levels of activating histone marks and low levels of repressive ones. Furthermore, ecDNA indicated a critical role in histone gene compensation [77, 78]. Previous research have revealed that ecDNAs improve active chromatin association over a distance, and ultra-remote chromatin contact has also been seen [79]. It is critical to gain a better understanding of the ecDNA-regulated signaling pathways to clarify the biological roles of ecDNA. For example, ecDNAs are responsible for increasing cancer cell proliferation as well as decreasing immune cell infiltration and action pathways [38]. Inflammation, oxidative stress, and the bystander effect could all be influenced by these mechanisms. EcDNA signaling can produce adaptive responses and bystander effects in the face of oxidative stress [80]. Apoptosis, oxidative stress, DNA change, ecDNA production, and subsequent alterations in bystander cells can all be caused by low-dose ionizing radiation. Bystander cells, like radiated cells, respond to oxidative stress by changing the shape of their nucleus, promoting actin polymerization, activating nucleolar organizer areas, and increasing the number of double-strand breaks [81–83]. The creation of ecDNA can stimulate pro-inflammatory cytokines, which are harmful to cancer cells. It was also indicated that ecDNA can significantly increase TLR9-MyD88-NF-kB signaling in the plasma of people with rheumatoid arthritis (RA), leading to the release of pro-inflammatory cytokines. Furthermore, it has been demonstrated that the high GC content of ecDNA has a replication-linked beneficial effect on the generation of pro-inflammatory cytokines [84]. EcDNAs have been shown to increase intra-tumor heterogeneity in a variety of cancers. The huge oncogene amplification in ecDNA, which results from asymmetric chromatin segregation during mitosis, aids cancer cells in adapting to their changing environment [42, 85]. EcDNA-encoded genes (e.g., MYC, MYCN, EGFR, Platelet-derived growth factor receptor alpha (PDGFRA), and epithelial transition (MET) are amplified in both primary and recurrent cancers, connecting the ecDNA to the evaluability of cancer cells under the selection pressure of the tumor microenvironment and therapeutic circumstance [85]. Further, it seems that the balance between ecDNA and HSR of chromosomes is critical in determining the evaluability of cancer cells. Since, ecDNA is more prevalent in tumors that are progressing and high levels of HSR are more frequently detected in tumors that are exposed to environmental factors [42]. It has been claimed that ecDNA can activate pathogen recognition receptors such as toll-like receptor (TLR), resulting in autophagy and apoptosis suppression. In line with these findings, cell-free and ecDNA-containing DNA has been found to govern autophagy in a TLR9-dependent manner [86]. Moreover, ecDNA transport via micronuclei or extracellular vehicles (EVs) has been shown to activate autophagy, which increases cancer cell survival in response to chemotherapy [86, 87]. These studies show that ecDNA influences cancer cells' drug sensitivity. Recently, it was discovered that ecDNA plays a role in increased metastasis and poor patient outcomes. In cancer cases with metastases, the level of ecDNA is significantly higher [88, 89]. EcDNAs transit between the nucleus and the cytoplasm in a mechanical way. They're frequently encased in micronuclei or shuttled around in EVs. These features aid ecDNA traversal of the cell membrane or exosomal trafficking to the extracellular area [10, 90]. As a messenger, ecDNA can be used by cancer cells to send oncogenic information to satellite tumors or other types of cells in the microenvironment. EcDNAs may drive both autocrine and paracrine signaling, promoting invasiveness, chemo-resistance, and the acquisition of a cancer stem cell-like phenotype [90]. EcDNA is linked to invasive tumor growth in many neuroblastomas, notably those expressing the MYCN oncogene [91]. Furthermore, the occurrence of DMs can be viewed as a recurrent and overlapping secondary alteration, which occurs most frequently in metastatic lesions [92]. In the subsequent malignant effusion of OC [92], malignant fibrous histiocytoma in bone, DMs are frequently observed [93], recurrent GBM tumors, and metastatic ASML mutations [94]. In comparison to the main tumor, all of these cancers show significant structural and numerical anomalies. However, the chromosomal morphology of metastatic cancers is poor, but oncogene amplification in ecDNA is high [93, 94]. Oncogenes on ecDNAs, such as MYC, increase tumor cell invasiveness and increase the amount of extrachromosomal DNA in metastatic tumor cells. These data support the hypothesis that ecDNA plays a role in tumor spread and invasion [95]. Senescence works as a powerful barrier to prevent normal cells from transforming into cancerous cancer cells. It's worth noting that daughter cells with lower ecDNA levels live longer than mother cells with higher ecDNA levels. Furthermore, ectopic expression of ecDNA sequences with autonomous replication can trigger cell cycle arrest, cell death, and inflammation associated with aging. The underlying processes by which ecDNA can aid malignant transformation by circumventing the senescence barrier, however, are still unknown [38, 96].

## Antitumor immunity

Encapsulating ecDNA formations within micronuclei, removing chromosomal H2AX foci, and enhancing immune responses are all necessary methods to destroy the formations. Micronuclei are generated by ecDNAs obtained from anaphase chromosomes after HU treatment, according to Shimizu and colleagues [97]. The formation of aneuploidy cells with increased viability is aided by these micronuclei [98]. In NB, ecDNA-containing micronuclei with amplified MYCN sequences have been found in in vivo studies [99]. Micronuclei’s DNA is prone to cytosolic release. The dynamics of the interplay between extracellular DNA and micronuclei can develop antitumor immune responses. Previous research has demonstrated that removing micronuclei reduces the number of cDNA genes carried by ecDNA and produced from colorectal and neuroectodermal tumor cells. This decrease would result in a tumor’s multiplication and malignancy lowering [100, 101]. As a result, micronuclei have been identified as a possible indicator of inflammation and DNA damage. Innate immunological responses, such as the activation of cGAS-STING innate immune signaling, are also thought to be triggered by them. As a result, research into a possible crosslink between ecDNA and antitumor immunity appears to be unavoidable [100, 101]. In the following sections, we will go over the most recent studies on our understanding of the biological activities of ecDNA in various malignancies.

## Role of cDNA in glioma cancer

**Fig. 2:**
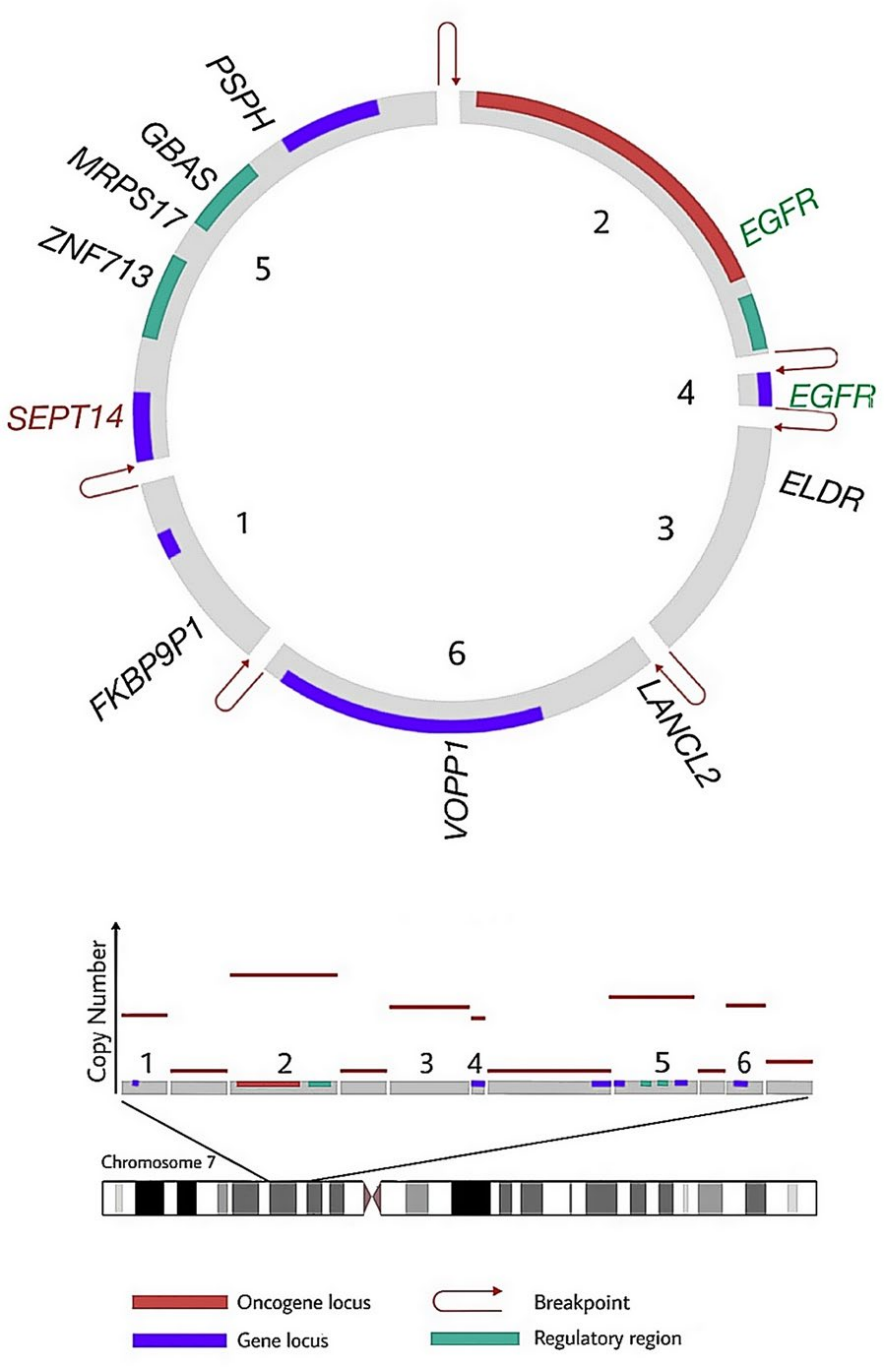
Important oncogene in ecDNA in glioblastoma [2]

Glioblastoma multiforme (GBM) is a type of highly aggressive and fatal primary brain tumor. The tumors have a wide level of inter- and intra-heterogeneity tumors which cause to decrease the efficient of treatment. The existence of tumor-initiating stem cells, as well as genetic and molecular differences, contribute to the heterogeneity of glioblastoma. EcDNA has recently been identified as a key component in glioblastoma heterogeneity and pathogenesis [39, 102]. However, in the past, data of neuroblastoma karyotype and pediatric brain tumor cells showed tiny ecDNA fragments existence. Thus, the biosynthesis and oncogenesis of ecDNAs, as well as their localization, putative link with extracellular vesicles, and their applicability in glioblastoma diagnostics and therapy, should be declared [103]. To screen and confirm the preclinical therapeutic target in this regard, tumor modeling using cell culture and orthotopic xenografts is imperative. The amplicon architect technique, which was recently developed, allows researchers to detect ecDNAs based on sequencing reads connecting amplified DNA segments [104]. Oncogenes found in ecDNA elements from glioblasvtoma patients included the MYC gene family (Table 2). Morton et al. recently showed that oncogenic transcriptional regulation is mediated by the enhancer architecture of ecDNA. They also suggested that during the creation of ecDNA, local enhancer elements are usually invariably incorporated with oncogenes in the ecDNA structures [25]. This discovery explained why oncogenes linked to ecDNA are so heavily transcribed. There is evidence that secondary somatic changes, including point mutations and insertion and deletions, can arise on ecDNAs, implying that glioblastoma has a constantly altering trajectory in original tumors, treatment, and relapse [1]. Uneven ecDNA inheritance in daughter cells allows for a rapid increase in genomic heterogeneity during gliomagenesis, independent of chromosomal DNA changes. De Carvalho et al. demonstrated that ecDNA exhibits different inheritance patterns and clonal selection dynamics between offspring cells as a result of comprehensive genomic and transcriptomic analysis of thirteen GBM tumor samples, neurosphere-forming cultures, and orthotopic xenograft models derived from these samples, which further support the notion that GBM genomic heterogeneity increases rapidly by implying even inheritance of ecDNA between offspring cells [85]. Two syngeneic GBM cultures that differed by whether or not they contained EGFR-encoding DMs were compared for invasiveness, heterogeneity, and radioresistance. However, more study is needed to determine whether the existence of EGFR-coding DMs or EGFR overexpression causes these findings [16]. Jonathanson et al. discovered that an epidermal growth factor receptor vIII (EGFRvIII) mutant ecDNA could be reversibly suppressed to prevent being targeted with inhibitors of the receptor. EGFR inhibitors were observed to reduce tumor cells with high expression of EGFRvIII in vitro and in vivo, but the cells rebounded when the treatment was discontinued, beyond the classical genetic explanations. Afterward, by observing cells in metaphase, they discovered a putative mechanism by which EGFRvIII nearly entirely amplified on ecDNA. additionally, EGFR inhibitors lowered the frequency of ecDNAs containing EGFRvIII, which then reemerged 1–2 weeks after drug removal [43]. To explain the above observation, Nikolaev et al. proposed a concept dubbed amplification-linked extrachromosomal mutations (ALEMs). The ALEMs are a form of cancer variation that originates in the extrachromosomal region with the ability to remove cancer cells. The idea is according to the fact that proliferation-promoting oncogenes are found on ecDNA, which enhances the chance of mutation. Based on exome sequencing of seven GBM patients, ALEMs are found not only in EGFR but also in PDGF R and Erb-B2- receptor tyrosine kinase 2 (ERBB2). In addition, an investigation of 4198 tumors revealed the existence of ALEMs in a variety of tumor types, implying that ALEMs may be the basic agents in therapy resistance in a variety of tumor types [105]. During the synthesis of ecDNA, enhancers are located in noncoding regions of the oncogene EGFR that are outside the TAD of the original chromosome, which might signify that ecDNA may be capable of establishing ultra-long-range chromatin interactions. These enhancers are rewired in ecDNA to increase oncogene EGFR expression and tumor fitness using CRISPR interference to disable individual enhancer activity revealed a unique mechanism of enhancers in controlling oncogene amplification [25]. EGFR was increased on ecDNA in glioblastoma cells, and glioblastoma cells carrying ecDNA had higher invasive characteristics and radiation resistance, according to Zhou et al. [16]. Researchers discovered that EGFR, c-MYC, N-MYC, and other genes depend on a large number of ecDNA amplification copies in glioblastoma cells. TKI resistance is exacerbated when ecDNA that encodes EGFRvIII is lost. A large proportion of glioblastoma cells resurfaced after stopping treatment with EGFR tyrosine kinase inhibitors (EGFRvIII ecDNA) in patient-derived models and samples from patients with glioblastoma [43]. Oncogenes including EGFR, PDGFRA, ERBB2, and Proto-Oncogene tyrosine-protein kinase (KIT) are found on ecDNA in glioblastoma and are amplified in huge numbers that have a key role in cancer promotion, according to research [105] (Fig. 2).

**Table 2:** Different oncogenes present on ecDNA and their functions

Cancer type	Associated gene(s) on ecDNA	Function	Refs.
Glioblastoma	*MYC*, *EGFR*, *PDGFRα*, *ERBB2*, *CDK4*, *MDM2*, *KIT*, *MET*	Increasing tumor invasiveness, radiation resistance, and drug resistance by upregulating a variety of oncogenes. In some cases, EGFRvIII and MDM2 amplification leads to Erlotinib resistance	[9, 43, 66, 90, 108]
Colon	*DHFR*, *c-MYC*, *BRCA1*	Silencing BRCA1 gene decreased the number of DM-amplified oncogenes and the number of DM copies in ecDNA by down-regulating DHFR. In addition, MTX-resistant cells containing DM increased susceptibility to MTX	[9, 41, 71, 109, 110]
Neuroblastoma	*MYCN*	The chromosomal genome needs to be remodeled, amplified, TERT stimulated, DCLK1 inhibited, and the presence of MYCN eliminated on ecDNA to increase HU sensitivity	[75, 99]
Cervical	*DHFR*	Promoting MTX resistance by *DHFR* amplification	[111]
Ovarian	*MYCN*, *EIF5AR*, *CA125*	Decreased levels of ecDNA-form *CA125* after HU	[66, 108, 112]
Breast	*DHFR*, *HER2*	Induced resistance to MTX by DM-form amplified DHFR is not affected by the loss of HER2 on ecDNA and trastuzamab therapy	[9, 73, 113]
Leukemia	*c-MYC*	Drug sensitivity ptomotion by down-regulating the *c-MYC*	[114]
Oral squamous cell carcinoma	*MDR1*	Enhancing HU sensitivity by Loss of *MDR1*	[9, 115]

## Role of ecDNA in NB cancer

N-MYC gene is discovered in ecDNA of neuroblastoma cancer by Kohl et al. [5]. It was also the first time that an oncogene was found in ecDNA. Following that, a thorough map of extrachromosomal DNA circularization in neuroblastoma cancer was created, as well as confirmation that the N-MYC gene was amplified on ecDNA in neuroblastoma cancer. In addition, short-read and nanopore sequencing were utilized to examine N-MYC amplicons structures. ChIP-seq, ATAC-seq, and high-throughput chromosomal conformation capture (Hi-C) were also employed to examine the chromatin. The first type is needed for the amplification of proximal enhancers, which controlled by the noradrenergic core regulatory circuit (CRC). The second type has no essential local enhancers; however, it has CRC-driven enhancers in remote chromosomal regions. As a result, the hijacking of ectopic enhancers can make up for the lack of local gene regulative elements and account for a remarkable portion of the structural change seen in N-MYC amplification [8]. Simultaneously, ecDNA dramatically boosted the oncogene telomerase reverse transcriptase (TERT) expression. Interestingly, ecDNA dramatically decreased the level of the tumor suppressor gene doublecortin-like and CAM kinase-like 1 (DCLK1) expression and it was initially identified in NB cell metaphase spreads [24]. Marking the first study to establish the presence of oncogenes in ecDNA. Ambros et al. looked at the NB cell lines with DMs in-depth and revealed that when exposed to low dosage e HU, the cells greatened and flattened their shape. Also, their granularity was increased and expressed senescence-associated-galactosidase (SA-GAL). All of these changes are senescence signs. It can be concluded that low-dose HU appears to be an efficient senescence promoter for tumor cells containing DMs [69]. Circular DNAs, such as ecDNA, eccDNA, and neochromosomes, have been discovered to modify the chromosomal genome in an unanticipated way. The remodeling is caused by circular DNA being generated and then reintegrated into chromosomal genomic loci, changing chromosomal gene expression, including oncogene and tumor suppressor expression. The oncogene TERT’s near circle integration is related to increased expression, while the tumor suppressor DCLK1's integration of circle fragments is associated with decreased expression. In cancer patients harboring ecDNA results in tumor reversion. Using FISH with a MYCN-specific probe, Ambros et al. observed spontaneous removal of extrachromosomal amplified MYCN in F-cells with phenotypic and evolutionary characteristics of malignant cells. As a result, it's thought that removing extrachromosomally amplified MYCN in NB is linked to tumor cell reversion [106].

## Conclusion

EcDNAs are circular chromatin elements found outside of the chromosome that usually carry oncogenes. EcDNAs have recently been discovered to be frequent in primary malignancies, indicating that they have a legitimate mechanism and adaptive reservoir for oncogene amplification. Through uneven segregation, ecDNAs can accumulate in cancer cells, giving them a competitive edge in response to selecting forces in the tumor microenvironment and cytotoxic treatment drugs.

The therapeutic value of analyzing ecDNAs of distinct regulatory sequences in the lack of oncogene amplification in both primary tumors and multiple cancer models will be enormous to detailed characterization of ecDNA targeted chromatin interactomes in malignancies. An imaging-based analysis is one of the ways currently used to characterize ecDNA or structural analysis of CN gain regions. Direct detection of intrachromosomal contact frequencies by chromatin interaction assays for instance ChIA-PET or Hi-C, in comparison to these approaches, provides an unbiased approach to uncovering ecDNA signals of various sizes, CNs, or sequence contexts. Considering the profile of ecDNA-associated chromatin interactomes and their target genes in primary tumor specimens, it seems that novel clinical applications can be provided by optimization protocol such as reducing required input cell numbers with along the combination of fluorescence-activated cell sorting to enrich transfected tumor cells.

## Data Availability

The datasets used and/or analyzed during the current study are available from the corresponding author on reasonable request.

## Abbreviations

EccDNA: Extrachromosomal circular DNA elements
EcDNA: Extrachromosomal DNA
WGS: Whole-genome sequencing
NB: Neuroblastoma
DMs: Double minutes
SEM: Scanning electron microscopy
HSR: Homogeneously staining regions
GBM: Glioblastoma
OC: Ovarian cancer
AML: Acute myeloid leukemia
SCLC: Small cell lung cancer
GLC1: SCLC cell line
SVs: Structural variations
BFB: Breakage-fusion-bridge cycle
IR: Replication initiation region
chrDNA: Chromosomal DNA
ATAC: Assay of transposase accessible chromatin
FISH: Fluorescence in situ hybridization
NGS: Next generation sequencing
TGS: Third-generation sequencing
ChIP: Chromatin immunoprecipitation
H3K4me1: Histone H3 at lysine 4
H3K27ac: Acetylation of histone H3 at lysine 27
PLAC seq: Proximity ligation assisted ChIP seq
FCNA: Focal copy number amplification
DAPI: 4,6-Diamidino-2-phenylindole
ChIA-PET: Chromatin interaction analysis by paired-end tag sequencing
MTX: Methotrexate
DHFR: Dihydrofolate reductase
MYC: Myelocytomatosis
NMYC: N-MYC proto-oncogene
RTK: Receptor tyrosine kinase
EGFR: Epidermal growth factor receptor
TLR: Toll-like receptor
EVs: Extracellular vehicles
PDGFRA: Platelet derived growth factor receptor alpha
MET: Mesenchymal to epithelial transition gene
CDK: Cyclin-dependent kinase
MDM2: Mouse double minute 2 homolog
ALEMs: Amplification-linked extrachromosomal mutations
ERBB2: Erb-B2 receptor tyrosine kinase 2
KIT: Tyrosine-protein kinase
Hi-C: High-throughput chromosomal conformation capture
CRC: Noradrenergic core regulatory circuit
TERT: Telomerase reverse transcriptase
DCLK: CAM kinase-like 1
SA-GAL: Senescence-associated-galactosidase
